# The relationship between proactive health management, perceived social support, and symptoms in perimenopausal women: a latent profile and mediation analysis

**DOI:** 10.3389/fpubh.2026.1889102

**Published:** 2026-09-29

**Authors:** Ying Yuan, Xuyan Liu, Tingting Ruan, Lijia Hou, Yiwei Li, Fan Zhang

**Affiliations:** 1 Department of Gynecology, De Yang People’s Hospital, Deyang, Sichuan, China; 2 Department of Public Health and Preventive Medicine, School of Medicine, Jinan University, Guangzhou, China

**Keywords:** latent profile analysis, perceived social support, perimenopausal symptoms, perimenopausal women, proactive health management

## Abstract

**Background:**

Perimenopausal women experience various physical and psychological symptoms. Proactive health management and perceived social support are considered important factors in alleviating these symptoms. However, previous studies have largely overlooked the heterogeneity of proactive health management among individuals. This study aims to identify latent profiles of proactive health management in perimenopausal women and to explore the mediating role of perceived social support between different levels of proactive health management and perimenopausal symptoms.

**Methods:**

Between February 2025 and August 2025, a total of 491 perimenopausal women were recruited from four tertiary hospitals in Sichuan Province. Data were collected using the perimenopausal women’s proactive health management scale, the Perceived Social Support Scale, and the Menopause Rating Scale. Latent profile analysis and mediation analysis were performed.

**Results:**

Three latent profiles of proactive health management in perimenopausal women were identified through latent profile analysis (LPA): the low-level passive health group (39.5%), the low-ability high-driven group (24.6%), and the high-level proactive health group (35.9%). Significant differences in perceived social support and perimenopausal symptoms were found between the low-level passive health group and the other groups. Mediation analysis revealed that the indirect association of perceived social support between the high-level proactive health group and perimenopausal symptoms was significant compared with the low-level passive management group (*β* = −1.389, SE = 0.451, 95% CI [−2.286, −0.504]), with a mediation proportion of 83.62%. In contrast, the indirect association for the low-ability high-drive group was not significant (*β* = −0.838, SE = 0.516, 95% CI [−1.822, 0.231]).

**Conclusion:**

There is heterogeneity in the level of proactive health management among perimenopausal women. Perceived social support shows a significant mediating association between the high-level proactive health management and perimenopausal symptoms. This mediating association was not observed in the low-ability high-driven group. It is recommended that community-based healthcare service systems and families pay attention to individual differences in health management among perimenopausal women. Beyond improving health literacy and self-management skills, health promotion programs should also leverage family and community support networks. Stratified and precise health promotion and intervention strategies should be developed for different subgroups to alleviate perimenopausal symptoms, thereby helping women transition through the perimenopausal period more smoothly and successfully.

## 1 Introduction

The acceleration of global population aging is projected to result in over 1.2 billion perimenopausal women worldwide by 2030. Enhancing self-health management capabilities among perimenopausal women can help prevent long-term health risks, reduce disease burden, and alleviate pressures on older adult care. Consequently, health management during the perimenopausal period has become a key focus in the field of public health (1, 2). The perimenopause is a critical physiological stage during which women transition from the reproductive period to old age, characterized by declining estrogen levels, which represent the primary biological mechanism underlying the emergence of physical and psychological symptoms (3–5). Approximately 80% of women are affected by perimenopausal symptoms, including vasomotor symptoms, sleep disturbances, mood swings, cognitive changes, and urogenital issues. Some symptoms persist for several years, significantly impairing physical function and quality of life (6–8). In addition to endocrine factors, symptoms occurrence and severity are closely associated with health behaviors, psychological regulation capacity, and social support (9). However, the relationships among proactive health management, perceived social support, and symptom severity in perimenopausal women remain unclear, and few studies have examined the role of perceived social support in the pathway between proactive health management and perimenopausal symptoms. Elucidating these relationships could provide precise targets for intervention, offer insights into the mechanisms underlying proactive health management, and carry important implications for public health.

Proactive health management refers to an individual’s proactive behaviors in acquiring health information, monitoring health risks, and engaging in health promotion during the health management process (10) and has received widespread attention in recent years. Proactive health management has been shown to help reduce symptom perception and improve quality of life across different populations (10, 11). Studies indicate that health management among perimenopausal women is generally passive, primarily due to a lack of health knowledge, low health literacy, and weak health empowerment (12–14). Research also shows that women with higher levels of proactive health management are more likely to adopt healthy lifestyles, maintain regular exercise, and employ appropriate coping strategies, thereby alleviating adverse symptoms (15–17). Thus, proactive health management may be a protective factor against perimenopausal symptoms. Perceived social support refers to individuals’ perceptions of receiving multidimensional social support sources and adequacy from family members, friends, and a significant other (18). Perimenopausal women in China generally have low levels of social support, and lower social support is significantly associated with higher symptom perception and negative emotional experiences (19). The underlying reason is that individuals with higher perceived social support are often better able to utilize family and social resources, seek help and support in a timely manner, and thereby relieve symptoms and improve quality of life (20, 21). Current studies suggest positive roles of proactive health management and perceived social support in alleviating symptoms among perimenopausal women, but few empirical studies have examined the relationships among these three factors. Previous research has mostly reflected health management capabilities through overall health behavior levels, yet overlooked the multidimensional nature of proactive health management. Moreover, when assessing proactive health management levels, most studies have taken a population-level approach, ignoring inter-individual heterogeneity. Therefore, this study attempts to employ a person-centered latent profile analysis (LPA) to accurately classify health management levels in perimenopausal women and to explore the potential mediating association of perceived social support in the relationship between latent classes of proactive health management and perimenopausal symptoms, based on cross-sectional analyses.

The Stress Process Model (SPM) elucidates the relationships among stressors, resources, and health outcomes. Stressors do not directly determine health outcomes but rather exert indirect effects through mediating pathways involving personal resources and coping behaviors (22, 23). Perimenopausal women experience multiple physiological, psychological, and social changes that collectively constitute persistent stressors. Within this framework, proactive health management can be conceptualized as an individual’s proactive coping behavior during multidimensional transitional stress, whereas perceived social support is regarded as an important stress-buffering resource. Stressors ultimately affect the health outcome—perimenopausal symptoms—by mobilizing or depleting social support resources. Social cognitive theory (SCT) further complements the micro-mechanisms of behavior–environment interaction. This theory posits that individuals acquire and maintain health behaviors through dynamic interactions among personal factors, behavioral patterns, and environmental influences, which are reciprocally determined (24, 25). Specifically, an individual’s proactive engagement in health management behaviors significantly increases the frequency and quality of positive interactions with the external environment (e.g., healthcare providers, family members, peer groups), thereby enabling the individual to obtain richer informational and emotional support from these social relationships. In other words, proactive health management is not only a behavioral manifestation of coping with stress but also an important pathway for activating and accumulating social support resources. Based on the integration of the above theoretical perspectives, this study constructs the following theoretical hypothesis pathway: the higher the level of proactive health management in perimenopausal women, the greater the frequency and quality of social interactions involved in processes such as health information seeking, self-monitoring of health risks, and planning of healthy lifestyles. Consequently, higher levels of social support are accumulated as stress-buffering resources, which in turn alleviate the severity of perimenopausal symptoms by attenuating negative physiological and psychological impacts.

Based on the current research status and theoretical foundations described above, this study aims to identify latent classes of proactive health management among perimenopausal women and, on that basis, reveal the factors influencing different classes. Furthermore, a mediation model is constructed to explore the role of perceived social support between different latent classes of proactive health management and perimenopausal symptoms, thereby providing empirical evidence for stratified and precise health management during the perimenopausal period. The following hypotheses are proposed, as illustrated in Figure 1:

1. There is population heterogeneity in the level of proactive health management among perimenopausal women.
2. Perceived social support and symptoms differ across different classes of proactive health management in perimenopausal women.
3. Perceived social support mediates the relationship between different latent classes of proactive health management and symptoms in perimenopausal women.

**Figure 1:**
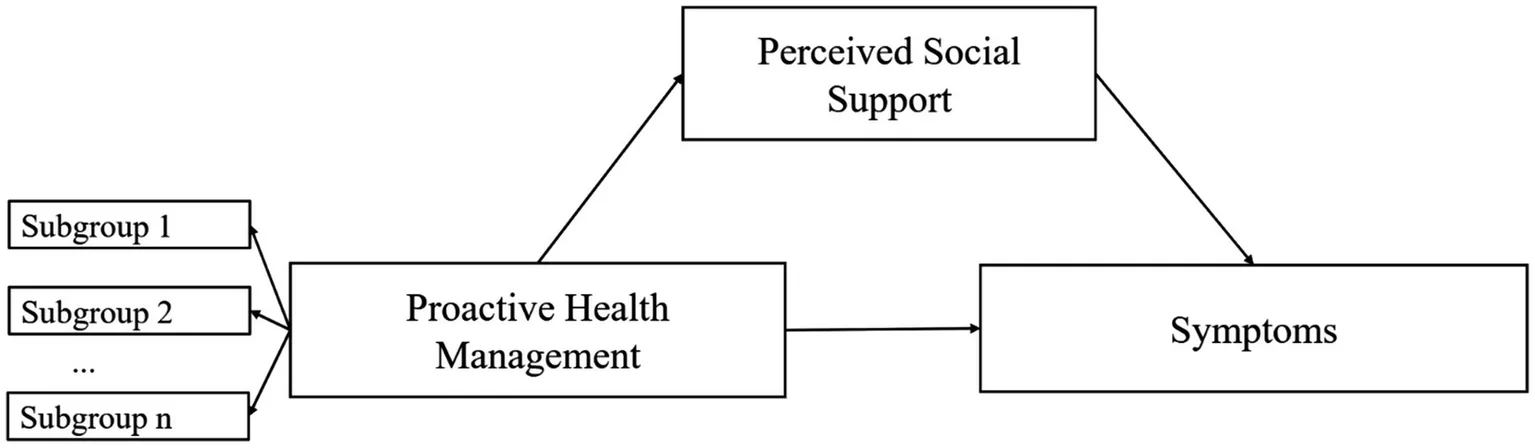
The hypothetical framework of proactive health management, perceived social support, and symptoms among perimenopausal women.

## 2 Methods

### 2.1 Study design and participants

This study employed a cross-sectional design, from February to August 2025, 544 perimenopausal women were recruited using a convenience sampling method from the gynecology and perimenopausal specialty outpatient clinics of four tertiary hospitals in Sichuan. The four hospitals were purposively selected to represent different geographic regions (e.g., provincial capital, prefecture-level cities, and county-level cities) within Sichuan Province, aiming to capture a certain degree of regional diversity. Inclusion criteria: (1) aged 40–60 years; (2) perimenopausal status was defined according to the STRAW+10 criteria, which encompassed the menopausal transition (stages −2 to −1) and early postmenopause (stage +1a). Operationally, this included women with menstrual cycle changes (prolongation or shortening) of >7 days, or those with amenorrhea for less than 12 months. (26); (3) providing informed consent and able to complete the questionnaire adequately. Exclusion criteria: (1) communication difficulties due to cognitive impairment, mental illness, hearing or visual abnormalities, or other factors; (2) severe physical illnesses such as malignant tumors; (3) non-natural menopausal status (e.g., due to surgery, radiotherapy, or chemotherapy); (4) experiencing major stressful life events (e.g., bereavement, divorce, significant property loss) within the past 6 months.

Sample size was calculated according to Kendall’s principle, which recommends that for a cross-sectional study, the sample size should be 10–20 times the number of independent variables (27). The demographic questionnaire used in this study included 12 variables, the Perceived Social Support Scale included 3 dimensions, the symptom burden scale included 4 dimensions, and the proactive health behavior scale included 4 dimensions, yielding a total of 23 variables. Therefore, the required sample size was estimated to be between 230 and 480 participants. To further enhance the robustness of the study, we considered the possibility of invalid questionnaires and assumed an invalid response rate of 10%. Based on this adjustment, the final target sample size was calculated as *n* = (230 ~ 480)/(1 − 10%) = 256 ~ 534 cases. Nylund-Gibson and Choi (28) recommended a minimum adequate sample size of 300 for latent profile analysis (LPA). Accordingly, based on the above criteria and accounting for the probability of invalid questionnaires unrelated to the study, 544 questionnaires were distributed, and 491 valid questionnaires were returned, meeting the sample size requirement.

This study adopted a cross-sectional design. The reporting followed the Strengthening the Reporting of Observational Studies in Epidemiology (STROBE) checklist. The study was approved by the Ethics Committee of Deyang People’s Hospital (approval no. 2024-4-125-K01), and informed consent was obtained from all participants.

### 2.2 Measurement

#### 2.2.1 Demographic characteristics

This study used a self-developed online questionnaire to collect demographic data and personal information items, including age, education level, place of residence, marital status, occupation, body mass index (BMI, kg/m^2^), type of medical insurance, menstrual status, average monthly household income per capita, chronic diseases, etc.

#### 2.2.2 Evaluation of perimenopausal symptoms

Perimenopausal symptoms were assessed using the Menopause Rating Scales (MRS), which were originally developed by Heinemann at the Center for Epidemiology and Health Research in Berlin (29) to evaluate the severity of menopausal symptoms and their impact on quality of life. The scale consists of 11 items, divided into three domains: somatic symptoms, psychological symptoms, and urogenital symptoms. The somatic symptoms domain includes four items (hot flashes/sweating, cardiac discomfort, insomnia, and joint/muscle pain); the psychological symptoms domain includes four items (depressed mood, irritability, anxiety, and physical and mental exhaustion); and the urogenital symptoms domain includes three items (decreased libido, bladder problems, and vaginal dryness). A Likert 5-point scale is used, with 0 = no symptoms and 4 = very severe symptoms. The total score is the sum of all items, ranging from 0 to 44 points. A total score of 0–4 indicates no symptoms, 5–8 indicates mild symptoms, 9–15 indicates moderate symptoms, and 16 or above indicates severe symptoms. Previous studies have confirmed that the MRS is a valid tool for evaluating perimenopausal symptoms in women and is suitable for Chinese perimenopausal women. In this study, the Cronbach’s *α* coefficient of the scale was 0.881.

#### 2.2.3 Evaluation of perceived social support

Perceived social support was assessed using the Perceived Social Support Scale (PSSS). This scale was originally developed by Zimet et al. (18) and later translated and revised by Jiang (30). It consists of 12 items covering three dimensions: family support, friend support, and other support. A Likert 7-point scale is used, with 1 = strongly disagree and 7 = strongly agree. The total score ranges from 12 to 84 points, with 12–36 indicating low support, 37–60 indicating moderate support, and 61–84 indicating high support. Higher scores reflect higher levels of perceived social support. In this study, the Cronbach’s *α* coefficient of the scale was 0.980.

#### 2.2.4 Evaluation of proactive health management

Proactive health management among perimenopausal women was assessed using the Proactive Health Management Scale for perimenopausal women, developed by Wang et al., (31). This scale is designed to comprehensively evaluate the behaviors and health management levels of perimenopausal women. The scale consists of 34 items divided into four dimensions: ability, support, motivation, and behavior. A Likert 5-point scale is used. For the ability dimension, the anchors range from 1 = completely unaware to 5 = completely aware; for the support, motivation, and behavior dimensions, the anchors range from 1 = completely inconsistent to 5 = completely consistent. All items are positively scored. The dimension score is calculated by summing the item scores within each dimension, and the total scale score is the sum of the four dimension scores. Higher scores indicate higher levels of proactive health management. In this study, the Cronbach’s *α* coefficient of the scale was 0.964.

### 2.3 Data collection

This survey was conducted face-to-face using the Wenjuanxing platform (https://www.wjx.cn), a widely used professional online survey website in China. The informed consent form and the questionnaire were imported into the WJX platform to generate an electronic link and QR code. After obtaining approval from the target hospitals, the questionnaire was distributed to all participants. Participants read the informed consent form before completing the questionnaire. The survey was anonymous, and confidentiality and voluntariness were ensured to maintain data reliability. The online questionnaire platform (Wenjuanxing) was configured to require responses to all items before submission, thus ensuring completeness of the collected data. Questionnaires were further screened to ensure high study quality by excluding those meeting the following criteria: (1) rapid response time (<2 min) or regular/repetitive answers—pre-survey results indicated that the time required to complete the questionnaire was approximately 5 min, thus <2 min was adopted as one of the censoring criteria; (2) contradictory sociodemographic characteristics.

### 2.4 Statistical analysis

All data in this study were analyzed using SPSS 27.0, Mplus 8.0, and R 4.5.1 software. First, descriptive statistics were calculated for all variables. Normally distributed data were expressed as mean ± standard deviation, while non-normally distributed data were expressed as median (interquartile range). Second, latent profile analysis (LPA) was used to explore subgroups of proactive health management. Most existing studies have adopted variable-centered analytical approaches, which have difficulty capturing potential heterogeneity in individual behavioral patterns. LPA, as a person-centered statistical method, can identify latent classes with characteristic consistency within a population, providing a basis for revealing differences in proactive health management patterns and developing precise intervention strategies. When performing LPA using Mplus 8.0, we started with a one-class model and sequentially tested one- to five-class models, comparing them based on fit indices. Lower AIC, BIC, and aBIC values indicate better model fit, while higher entropy values reflect more accurate class classification. An entropy value ≥ 0.8 was considered indicative of good classification quality. The Lo–Mendell–Rubin (LMR) test and the bootstrapped likelihood ratio test (BLRT) were used to evaluate changes in model fit when adding an additional class; a *p*-value < 0.05 indicated that the current class model was superior to the previous one. Third, after determining the optimal number of classes through LPA, multivariate logistic regression was used to compare differences in demographic characteristics among the three classes. Statistical tests were two-sided, and the significance level was set at 0.05. The BCH command analyzed the relationship between different categories of proactive health management and perimenopausal symptoms as well as perceived social support. Fourth, the mediation role of perceived social support between proactive health management and perimenopausal symptoms was analyzed using model 4 in the SPSS PROCESS 4.1 macro. The latent profiles of proactive health management (with the low-level passive management group as the reference) were used as the independent variable, perceived social support as the mediator, and perimenopausal symptoms as the dependent variable. Age, average monthly household income per capita, education level, and menstrual status were included as covariates in the mediation model. To maintain the simplicity of the model and avoid overfitting, we included only those variables that have been consistently identified in the literature as potential confounders as control variables (32, 33). The bootstrap method (34) was used to assess the significance of the indirect association, with focus on the 95% confidence interval; the number of resampling iterations was set to 5,000.

## 3 Results

### 3.1 Common method bias test

The Harman single-factor test was used to examine common method bias. Exploratory factor analysis was performed on all items. The unrotated matrix results showed that 12 factors had eigenvalues greater than 1, and the variance explained by the first factor was 34.45%, which is below the critical threshold of 40%, suggesting that no severe evidence of common method bias was detected.

### 3.2 Sample characteristics

A total of 544 perimenopausal women were enrolled as study subjects. After excluding invalid questionnaires with incomplete, rapid, and contradictory responses, 491 perimenopausal women completed the questionnaire assessment, yielding a response rate of 90.3%. The flow chart of the study is given in Figure 2.

**Figure 2:**
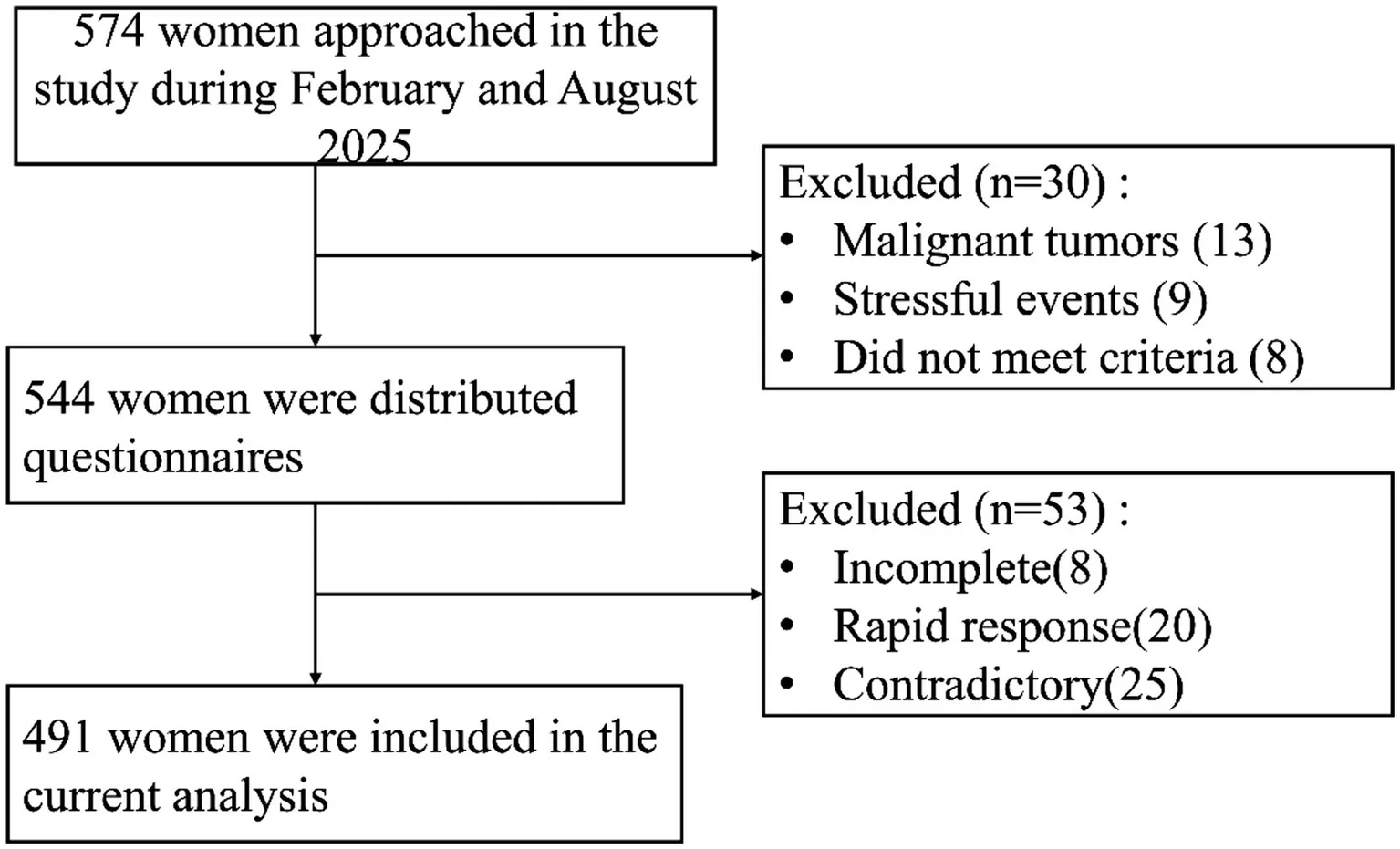
The flow chart of the study.

### 3.3 Descriptive analysis of general characteristics

Among the 491 perimenopausal women in this survey, the largest age group was 46–50 years (29.3%). The majority had an education level of high school or below (80.2%). Regarding place of residence, 42.4% lived in rural areas and 57.6% in urban areas. Most women had a normal body mass index (BMI) range (60.9%), followed by overweight (27.9%). In terms of menstrual status, the majority were in the early menopausal transition stage (48.1%), followed by early postmenopause (36.9%).

### 3.4 Correlation analysis of proactive health management, perimenopausal symptoms, and social support

The median (interquartile range) for each variable was as follows: proactive health management, 98 (75, 127); social support, 64 (53, 72); perimenopausal symptoms, 7 (3, 13). Spearman correlation analysis was performed on the three variables and their dimensions. The results showed that proactive health management was significantly positively correlated with social support (*r_s_* = 0.196, *p* < 0.001) and significantly negatively correlated with perimenopausal symptoms (*r_s_* = −0.186, *p* < 0.001). Perimenopausal symptoms were significantly negatively correlated with social support (*r_s_* = −0.534, *p* < 0.001).

### 3.5 Latent profile analysis of proactive health management

Latent profile analysis was performed on the 34 items of the Proactive Health Management Scale, with one- to five-class models being estimated. As the number of classes increased from 1 to 5, the AIC, BIC, and aBIC values gradually decreased, indicating progressive improvement in model-data fit; however, the reductions from the three-class to the four-class model were markedly attenuated. The LMR test *p* values for the four- and five-class models were 0.0187 and 0.5723, respectively, suggesting that adding a fourth or fifth class did not yield a significant improvement over the previous model. The LMR and BLRT tests for the two- and three-class models were both significant (*p* < 0.001), supporting the three-class solution over the two-class model. The three-class model had an entropy of 0.983, reflecting excellent discriminative ability and clustering performance. The class proportions in the three-class solution were 39.5%, 24.6%, and 35.9%, all sufficiently large and well-balanced, and the three classes were clearly interpretable in practical terms (Table 1). In summary, considering both parsimony and interpretability, the three-class model was selected as the optimal solution. Proactive health management was categorized into three classes: 39.5% of participants exhibited consistently low levels of proactive health management and were classified as the “low-level passive management group” (*N* = 194); 35.9% of participants exhibited consistently high levels and were classified as the “high-level proactive management group” (*N* = 176); and 24.6% of participants showed low levels in the ability dimension of proactive health management while scoring between low and high levels on the other three dimensions, and were classified as the “low-ability high-drive group” (*N* = 121), as illustrated in Figure 3.

**Table 1:** Latent profiling and naming of proactive health management traits.

Indicators	LPA model				
	1-Class	2-Class	3-Class	4-Class	5-Class
Fit statistics					
*k*	68	103	138	173	208
LL	−29127.586	−25629.842	−23971.106	−23290.748	−22744.030
AIC	58391.172	51465.685	48218.212	46927.495	45904.060
BIC	58676.53	51897.919	48797.322	47653.48	46776.921
aBIC	58460.699	51570.998	48359.311	47104.38	46116.731
Entropy	—	0.987	0.983	0.981	0.979
LMR	—	<0.001	<0.001	0.0187	0.5723
BLRT	—	<0.001	<0.001	<0.001	<0.001
Group size(%)					
C1	491 (100%)	296 (60.3%)	194 (39.5%)	187 (38.1%)	100 (20.4%)
C2	—	195 (39.7%)	121 (24.6%)	91 (18.5%)	91 (18.5%)
C3	—	—	176 (35.9%)	105 (21.4%)	89 (18.1%)
C4	—	—	—	108 (20%)	106 (21.6%)
C5	—	—	—	—	105 (21.4%)

*k*, Number of free parameters; LL, Log-likelihood; AIC, Akaike information criteria; BIC, Bayesian information criteria; aBIC, adjusted Bayesian information criterion; BLRT, Bootstrap likelihood ratio test; VLMR, Vuong–Lo–Mendell–Rubin test.

**Figure 3:**
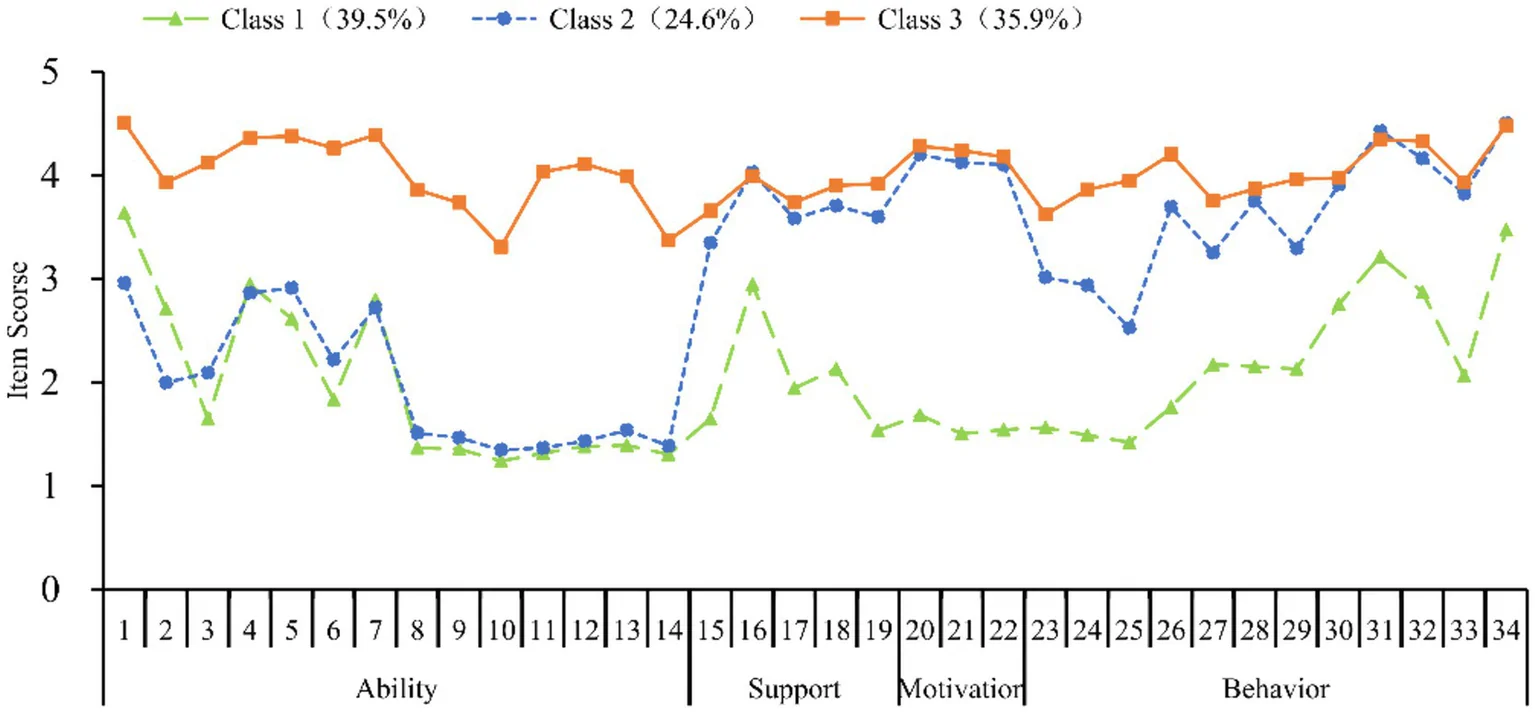
Latent profiles of proactive health management identified by latent profile analysis. Class 1: Low-level passive group; Class 2: Low-ability high-driven group; Class 3: High-level proactive health group.

### 3.6 Sociodemographic characteristics and differences among the three latent classes of proactive health management in perimenopausal women

The results of the univariate analysis showed that the distribution of perimenopausal women across the three latent classes of proactive health management differed significantly by age, education level, place of residence, chronic disease, menstrual status, occupation, and average monthly household income per capita (Table 2). Therefore, these variables were included as control variables in subsequent analyses.

**Table 2:** Demographic characteristics of the study population.

Variables	Class 1 (*n* = 194)	Class 2 (*n* = 121)	Class 3 (*n* = 176)	*χ*^2^ /H	*p*
Age (years), *n* (%)				39.683	<0.001
40–45	40 (29.9)	27 (20.1)	67 (50)		
46–50	70 (48.6)	34 (23.6)	40 (27.8)		
51–55	38 (28.6)	48 (36.1)	47 (35.3)		
56–60	46 (57.5)	12 (15)	22 (27.5)		
Education level, *n* (%)				54.508	<0.001
High school or below	177 (44.9)	105 (26.6)	112 (28.4)		
College	9 (26.5)	8 (23.5)	17 (50)		
Bachelor’s degree or above	8 (12.7)	8 (12.7)	47 (74.6)		
Residence, *n* (%)				30.502	<0.001
Rural	108 (51.9)	52 (25)	48 (23.1)		
Urban	86 (30.4)	69 (24.4)	128 (45.2)		
Marital status, *n* (%)				3.581	0.167
Married	169 (38.1)	111 (25.1)	163 (36.8)		
Unmarried	25 (52.1)	10 (20.8)	13 (27.1)		
BMI, *n* (%)				10.644	0.100
Underweight	10 (47.6)	5 (23.8)	6 (28.6)		
Normal	126 (42.1)	60 (20.1)	113 (37.8)		
Overweight	45 (32.8)	45 (32.8)	47 (34.3)		
Obese	13 (38.2)	11 (32.4)	10 (29.4)		
Chronic diseases, *n* (%)				13.011	<0.001
Yes	55 (53.4)	25 (24.3)	23 (22.3)		
No	139 (35.8)	96 (24.7)	153 (39.4)		
Menstrual status, *n* (%)				28.153	<0.001
Early perimenopause	71 (30.1)	61 (25.8)	104 (44.1)		
Late perimenopause	41 (55.4)	8 (10.8)	25 (33.8)		
Early postmenopause	82 (45.3)	52 (28.7)	47 (26)		
Occupation, *n* (%)				78.935	<0.001
Manual laborer	74 (46.8)	50 (31.6)	34 (21.5)		
On-duty staff	33 (35.1)	20 (21.3)	41 (43.6)		
Self-employed	33 (47.8)	19 (27.5)	17 (24.6)		
Professional/technical staff	4 (12.5)	1 (3.1)	27 (84.4)		
Unemployed/looking for work	8 (21.1)	15 (39.5)	15 (39.5)		
Full-time homemaker	30 (52.6)	12 (21.1)	15 (26.3)		
Retired	12 (27.9)	4 (9.3)	27 (62.8)		
Medical insurance, *n* (%)					0.159^*^
Insured	192 (40.2)	117 (24.5)	169 (35.4)		
Self-paid	2 (15.4)	4 (30.8)	7 (53.8)		
Monthly household *per capita* income, *n* (%)				87.249	<0.001
<4,000 RMB	124 (57.7)	52 (24.2)	39 (18.1)		
4,000–6,000 RMB	44 (30.3)	47 (32.4)	54 (37.2)		
>6,000 RMB	26 (19.8)	22 (16.8)	83 (63.4)		

*χ*^2^ = Chi-square test; H = Kruskal-Wallis test; *Fisher’s Exact Test. Class 1 = Low-level passive group; Class 2 = Low-ability high-driven group; Class 3 = High-level proactive health group.

### 3.7 Multifactorial analysis of the three latent classes of proactive health management

Prior to regression analysis, linear regression diagnostics were used to assess multicollinearity among the independent variables. The results showed that all variance inflation factors (VIF) ranged from 1.027 to 2.328, and tolerance values were all >0.2, indicating no significant multicollinearity. Using the latent class of proactive health management as the dependent variable, multinomial logistic regression was performed to explore the associations between sociodemographic and health-related factors and class membership. The results showed that older age, lower education level, lower income, rural residence, presence of chronic diseases, being a manual worker, early menopausal transition stage, and being a full-time homemaker were more likely to be associated with classification into the low-level passive management group. Further details are shown in Supplementary Table S1.

### 3.8 LPA-based proactive health management differences on perimenopausal symptoms and perceived social support

Comparing the three profiles revealed significant differences (*p* < 0.01) in perimenopausal symptoms and perceived social support between high-level proactive health group (C3) and both low-level passive group (C1) and low-ability high-driven group (C2). Specifically, C3 had significantly lower symptom levels but higher social support levels than C1 and C2, with no significant differences between C1 and C2 on either measure (see Table 3).

**Table 3:** Differential analysis of perimenopausal symptoms and perceived social support among individuals across three classes.

Variable	Overall *χ*^2^	p	C1 M ± SE	C2 M ± SE	C3 M ± SE	Post-hoc tests (*p*)			Significant pairwise comparisons
						C1 vs. C2	C1 vs. C3	C2 vs. C3	
Perimenopausal symptoms	13.924	0.001	9.607 ± 0.527	10.316 ± 0.829	7.348 ± 0.501	0.473	0.002	0.002	C1 > C3, C2 > C3
Perceived social support	21.678	<0.001	57.702 ± 1.244	59.741 ± 1.591	65.185 ± 1.101	0.316	<0.001	0.005	C3 > C1, C3 > C2

All means are BCH-weighted marginal means. Overall group differences were tested using BCH-adjusted Wald chi-square tests. Pairwise comparisons were based on BCH post-hoc tests, with statistically significant differences (*p* < 0.01). C1: Low-level passive group; C2: Low-ability high-driven group; C3: High-level proactive health group.

### 3.9 Mediation analysis

A mediation model was constructed with the latent classes of proactive health management among perimenopausal women as the independent variable, since Group C1 exhibited relatively balanced scores across all dimensions, it was designated as the control group, perceived social support as the mediator, and perimenopausal symptoms as the dependent variable, adjusting for age, income, education level, and menstrual status. When the independent variable has k categories (*k* ≥ 3), relative mediation analysis can be employed. The latent profiles of proactive health were dummy-coded, yielding two dummy variables (D1: 0 = Group C1, 1 = Group C2; D2: 0 = Group C1,1 = Group C3). As shown in Table 4, for the low-ability high-driven group (C2), the total association between proactive health management and symptoms was not significant (*β* = 0.014, SE = 0.854, 95% CI [−1.663, 1.692]), nor was the indirect association through perceived social support (*β* = −0.838, SE = 0.516, 95% CI [−1.822, 0.231]). For the high-level proactive health group (C3), the total association between proactive health management and symptoms was significant (*β* = −1.661, SE = 0.783, 95% CI [−3.200, −0.122]). After including perceived social support as a mediator, the direct association of C3 with symptoms was not significant (*β* = −0.273, SE = 0.640, 95% CI [−1.530, 0.985]), whereas the indirect association through perceived social support was significant (*β* = −1.389, SE = 0.451, 95% CI [−2.286, −0.504]). The indirect association accounted for 83.62% of the total association. These findings suggest that perceived social support is consistent with a full mediating role in the observed association between high-level proactive health management and perimenopausal symptoms. The standardized regression coefficients for each path in the model are shown in Figure 4.

**Table 4:** Direct and indirect associations of proactive health management latent classes with perimenopausal symptoms.

Group	Effect	SE	LLCI	ULCI	Proportion
Low-ability high-driven group (C2)					
Relative direct effect	0.852	0.693	−0.51	2.214	
Relative indirect effect	−0.838	0.516	−1.822	0.231	
Relative total effect	0.014	0.854	−1.663	1.692	
High-level proactive health group (C3)					
Relative direct effect	−0.273	0.640	−1.530	0.985	
Relative indirect effect	−1.389	0.451	−2.286	−0.504	83.62%
Relative total effect	−1.661	0.783	−3.200	−0.122	

Low-level passive management group is used as the reference. SE, standard error; LLCI, lower level of confidence interval; ULCI, upper level of a confidence interval.

**Figure 4:**
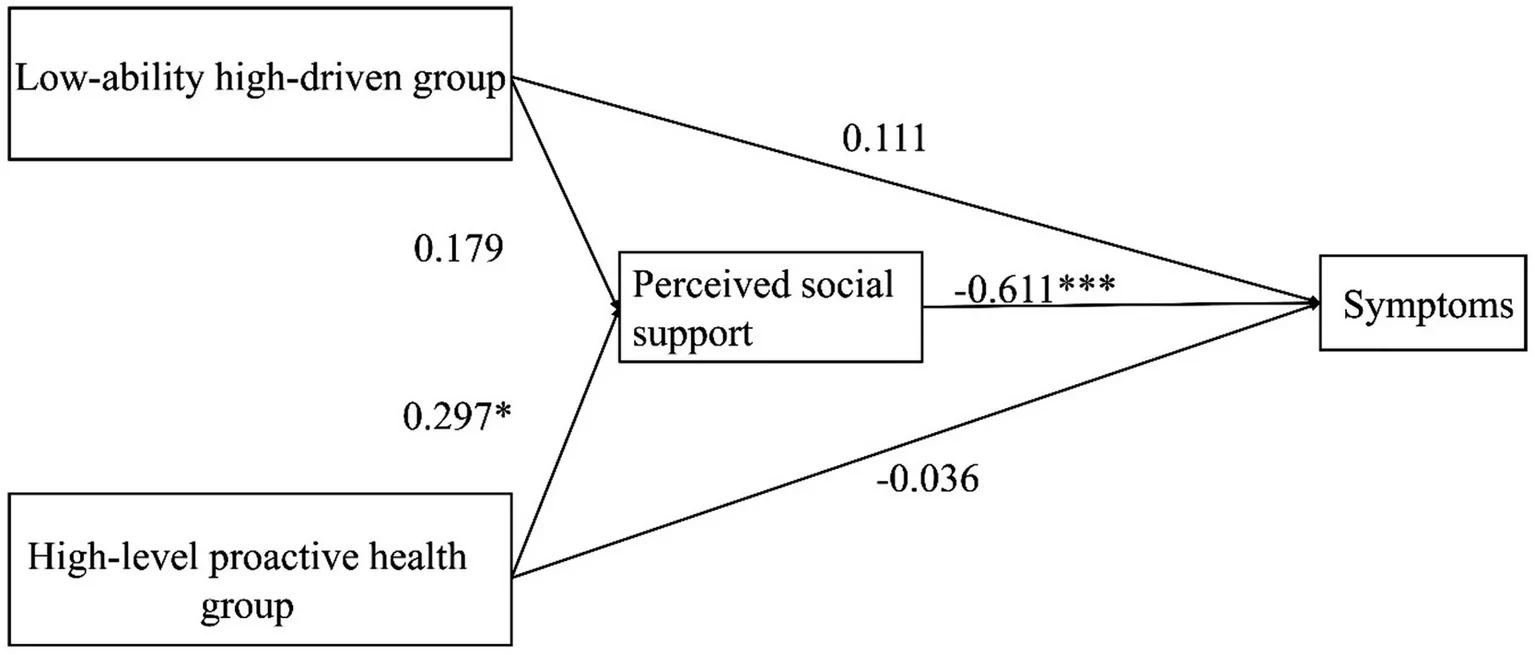
The mediating association path of perceived social support in the relationship between latent classes of proactive health management and symptoms among perimenopausal women. Low-level passive management group is used as the reference. Path coefficients are standardized (*β*). *p < 0.05, **p < 0.01, ***p < 0.001.

## 4 Discussion

### 4.1 Heterogeneity exists in the level of proactive health management among perimenopausal women

This study identified three latent classes of proactive health management in perimenopausal women using LPA, confirming H1. The low-level passive management group accounted for 39.5% of participants and scored low across all dimensions of proactive health management. The results of multinomial logistic regression showed that, compared with the high-level proactive management group and the low-ability high-drive group, factors such as older age, early menopausal transition stage, lower education level, presence of chronic diseases, being a manual worker, being a full-time homemaker, limited economic status, or rural residence were associated with belonging to the low-level passive management group, which is consistent with previous studies (32, 35, 36). Lack of health knowledge is closely related to passive coping behaviors. Individuals with low levels of proactive health management have insufficient health knowledge and self-management abilities, which impairs their capacity to cope with perimenopausal symptoms such as urinary incontinence and vaginal dryness (37). Limited accessibility to healthcare resources and poor health behavior capability further weaken their willingness to seek interventions. Lower proactive health management often leads to unhealthy behaviors and higher symptom burden (12, 38). Community health workers and healthcare providers should pay particular attention to perimenopausal women with low-level proactive health management, especially those who are older, in the early menopausal transition stage, have lower education levels, have chronic diseases, are manual workers or full-time homemakers, have limited economic status, or live in rural areas. It is recommended to implement a community-based health education approach (39), using plain language and educational materials and training activities that are culturally appropriate, to carry out a nurse-led digital education program (40).

The low-ability high-drive group accounted for 24.6% of participants. Women in this group scored low on the ability dimension of proactive health management but scored high on the other dimensions, indicating that improvement in their proactive health management level is associated with adequate utilization of external resources. This suggests that attention should be paid to external support for perimenopausal women (41). At the same time, enhanced intrinsic motivation may help stimulate women to actively participate in perimenopausal health management, thereby forming sustained and stable health behavior patterns (42). For this specific group, the focus should be on enhancing their capabilities, including targeted self-management training, setting behavioral goals, and developing problem-solving skills (43).

The high-level proactive management group (35.9%) scored high across all dimensions and had the mildest symptoms severity, indicating that proactive health management has significant advantages in alleviating perimenopausal symptoms. Previous studies have shown that high levels of health management capability in perimenopausal women are closely associated with relief of perimenopausal symptoms (44), which is consistent with the findings of the present study. Women with high levels of health behaviors typically have stronger abilities to obtain and understand health information, enabling them to recognize perimenopausal symptoms earlier and adopt effective self-management strategies in a timely manner, such as improving sleep quality, engaging in regular physical exercise, and making reasonable dietary adjustments, thereby effectively reducing symptom burden (17). For perimenopausal women with high levels of proactive health behaviors, it is recommended to implement maintenance strategies using a peer-support-based approach (45). Therefore, it is recommended that health interventions for perimenopausal women should focus on strengthening health beliefs and self-efficacy through tiered health education and behavioral intervention programs, stimulating intrinsic motivation for health behaviors, enhancing the ability to understand and apply health information, and promoting long-term maintenance of proactive health management to improve perimenopausal symptoms.

### 4.2 Relationships among proactive health management, perceived social support, and perimenopausal symptoms

This study found that proactive health management was positively correlated with perceived social support and negatively correlated with perimenopausal symptoms. Moreover, significant differences in social support and perimenopausal symptoms were observed across different latent classes, which is consistent with previous findings (19), thereby validating H2. This finding corroborates the longitudinal evidence reported by Xie et al. (46) on the heterogeneity of depressive symptoms among Chinese menopausal women. Higher levels of proactive health management were significantly associated with reduced perimenopausal symptoms, suggesting that proactive health management may be a factor related to perimenopausal symptoms (47). This study further examined the mediating role of perceived social support in the association between proactive health management and perimenopausal symptoms through mediation analysis in the cross-sectional context (H3). Mediating associations varied significantly across latent classes, and this differential pattern emerges as a key finding of this research. Compared with the low-level passive management group, in the high-level active health management group (C3), perceived social support fully mediated the relationship between proactive health management and perimenopausal symptoms; the mediating association accounted for 83.62% of the total association, and the indirect association was significant (*β* = −1.389, SE = 0.451, 95% CI [−2.286, −0.504]), while the direct association was not significant. These results indicate that for perimenopausal women with higher health information acquisition abilities, health behavior implementation capabilities, and intrinsic motivation, the association of proactive health management on symptoms improvement is almost entirely mediated through the perceived social support pathway. According to theoretical frameworks, proactive health management enhances perimenopausal women’s connections with external support; positive interactions enable them to obtain more emotional, instrumental, and informational support, thereby strengthening coping strategies for the perimenopause, which may allow them to identify symptoms early and respond in a timely manner, ultimately improving perimenopausal symptoms (48). In the low-capacity high-motivation group (C2), the mediating association of perceived social support was not significant (*β* = −0.838, SE = 0.516, 95% CI [−1.822, 0.231]), with the confidence interval crossing zero. Although women in this group subjectively reported high motivation and willingness to manage their health, they may fail to effectively translate their intentions into substantive health-related interactions or to acquire adequate social support, owing to insufficient health knowledge and limited skills in identifying and applying health information (49). This finding suggests that the mediating role of perceived social support varied across groups with different health management models, with full mediation observed only in the high-level proactive health group (C3). Specifically, this differential pattern appears to depend on whether individuals possess the fundamental competencies required to translate health management intentions into effective social engagement. Han et al. (50) provided a mechanistic account for this phenomenon, noting that Chinese patients frequently exhibit information avoidance behaviors in health-seeking contexts, driven by factors such as family caregiving burden, self-stigma, and psychological defense mechanisms. Consequently, some women may be unable to overcome capacity constraints despite a strong desire to improve their health, and thus fail to transform intrinsic motivation into proactive actions for accessing external resources. For the low-capacity, high-motivation group, the absence of a significant link between perceived social support pathways and health outcomes may therefore arise from this group’s insufficient foundational capacity to activate and effectively utilize social support resources.

Compared with the low-level passive management group (C1), the Low-ability high-driven group (C2) showed a positive correlation with symptoms, while the high-level proactive health management group (C3) showed a negative correlation with symptoms. This seemingly contradictory phenomenon suggests that “high motivation” divorced from a foundation of capacity not only fails to contribute to symptom relief but may even be associated with more negative health experiences. Although women in Group C2 held a positive attitude toward health management, their limited ability to access health information and lack of self-management skills restricted their capacity to translate their intentions into effective health behaviors, potentially leading to cognitive dissonance, reduced self-efficacy, and an increased psychological burden (51). Furthermore, the higher level of management motivation in Group C2 may stem from health anxiety rather than positive health beliefs; excessive focus on physical symptoms may, in turn, intensify the subjective perception of perimenopausal symptoms (52). This finding underscores that capacity building is as essential as motivation enhancement in health management interventions, and neither component alone suffices.

Thus, health management can indirectly improve physical and psychological experiences by building positive psychological resources and enhancing individuals’ perception of social support (53). The buffering role of perceived social support on symptom burden has been demonstrated in various populations, including alleviating postpartum depressive symptoms in women (54) and improving psychological symptoms in patients with inflammatory bowel disease (55). Therefore, it is recommended that community healthcare systems establish health records for perimenopausal women, provide health education (56), conduct regular follow-ups (57), improve awareness of the perimenopause, enhance family members’ nursing care, understanding, and psychological support for perimenopausal women, and expand access to resources and opportunities to broaden social networks. These efforts would enable women to proactively seek external support when they recognize perimenopausal symptoms early and utilize social support systems to alleviate physical and psychological symptom burdens. Based on the above findings, for perimenopausal women with insufficient health management capabilities, intervention strategies should not be limited to one-way transmission of health knowledge but should place greater emphasis on enhancing individuals’ ability to recognize, actively seek, and effectively utilize social support resources. Creating a supportive family and social environment, promoting peer interaction and social participation, and relying on continuous and accessible healthcare service systems can effectively facilitate the formation and maintenance of proactive health management behaviors in perimenopausal women, thereby reducing the burden of perimenopausal symptoms.

### 4.3 Limitations

This study has several limitations. First, the data were collected only from patients attending tertiary hospitals, which may have introduced selection bias, as these women might have higher health awareness or more severe symptoms than the general perimenopausal population, potentially limiting the generalizability of our findings. Second, this study employed a cross-sectional design, in which all variables were measured at the same time point, thus, the results reflect statistical associations among the variables and do not allow for examination of dynamic changes over time,cannot establish causal relationships among the three variables. Future longitudinal studies are needed to examine causality. Third, proactive health management and perimenopausal symptoms in perimenopausal women are influenced by multiple factors, which warrant further investigation in future research. Fourth, all variables in this study were measured using self-report questionnaires. Although Harman’s one-way test did not reveal any serious bias, we cannot completely rule out the possibility that common-method variance may have influenced the study results. Fifth, the subsequent intermediary analyses treated latent class membership as an observed variable, which may not fully account for classification uncertainty. However, the entropy value (0.983) and the average posterior probabilities for the three classes (0.995, 0.986, and 0.992) were all well above the recommended thresholds, indicating excellent classification accuracy and thus minimal influence of classification uncertainty on the results. We also attempted the BCH three-step correction method, but it proved infeasible due to excessive sample loss caused by invalid weights. Future studies with larger samples may consider employing for subsequent analyses.

## 5 Conclusion

This study reveals significant heterogeneity in proactive health behaviors among perimenopausal women, and significant differences in perceived social support and symptoms across different latent classes. Our findings indicate that perceived social support is linked to the relationship between proactive health management and perimenopausal symptoms, yet this linkage is not uniform across all women. Rather, it varies depending on how women approach health management. Among those who engaged in high-level proactive health management, social support fully accounted for the association with symptom alleviation, suggesting that these women may have drawn on and built up social resources as a pathway to better outcomes. In contrast, for women in the low-ability high-driven group, this indirect pathway was not evident. These women, though highly motivated, may lack the practical skills needed to convert their intentions into actual social support. Taken together, our results point to a conditional role of perceived social support: its protective function appears to depend on whether women have sufficient proactive health management capacity to mobilize social resources effectively. These findings deepen our understanding of perimenopausal symptoms interventions and contribute to the theoretical basis for risk stratification and targeted strategy design. In practice, interventions should be tailored to women’s health behavior profiles: strengthening health education to improve perimenopausal knowledge and self-management skills, enhancing support networks and access to care, and bolstering health beliefs and self-efficacy to encourage proactive health management and reduce symptom burden.

## Data Availability

The raw data supporting the conclusions of this article will be made available by the authors, without undue reservation.

## References

[1] Ilankoon IMPS Samarasinghe K Elgán C. Menopause is a natural stage of aging: a qualitative study. BMC Womens Health. (2021) 21:47. doi: 10.1186/s12905-020-01164-6, 33522914 PMC7849153

[2] Healthy China Initiative (2019) Healthy china action promotion committee issued the Healthy China Action (2019–2030). Available online at: https://www.nhc.gov.cn/guihuaxxs/c100133/201907/2a6ed52f1c264203b5351bdbbadd2da8.shtml (Accessed November 8, 2025)

[3] Traish AM Vignozzi L Simon JA Goldstein I Kim NN. Role of androgens in female genitourinary tissue structure and function: implications in the genitourinary syndrome of menopause. Sex Med Rev. (2018) 6:558–71. doi: 10.1016/j.sxmr.2018.03.005, 29631981

[4] Traish AM Botchevar E Kim NN. Biochemical factors modulating female genital sexual arousal physiology. J Sex Med. (2010) 7:2925–46. doi: 10.1111/j.1743-6109.2010.01903.x, 20626599

[5] Auro K Joensuu A Fischer K Kettunen J Salo P Mattsson H . A metabolic view on menopause and ageing. Nat Commun. (2014) 5:4708. doi: 10.1038/ncomms5708, 25144627

[6] Lee BG Ham OK Kim SH Lee EJ Kang HS Chae D. Concomitants of menopause-specific quality of life in premenopausal and post-menopausal women living in South Korea. Women Health. (2020) 60:887–98. doi: 10.1080/03630242.2020.1767262, 32466716

[7] Duralde ER Sobel TH Manson JE. Management of perimenopausal and menopausal symptoms. BMJ. (2023) 382:e072612. doi: 10.1136/bmj-2022-072612 37553173

[8] Simpson EEA Doherty J Timlin D. Menopause as a window of opportunity: the benefits of designing more effective theory-driven behaviour change interventions to promote healthier lifestyle choices at midlife. Proc Nutr Soc. (2024) 83:120–9. doi: 10.1017/S0029665123004810, 37955168

[9] Barati M Akbari-heidari H Samadi-yaghin E Jenabi E Jormand H Kamyari N. The factors associated with the quality of life among postmenopausal women. BMC Womens Health. (2021) 21:208. doi: 10.1186/s12905-021-01361-x, 34006264 PMC8130393

[10] Zhou X Zhang M Xie Y Zhao P Chang A Lian X . A mixed-methods systematic review of factors influencing health behaviors in stroke patients based on active health. J Nurs Sci. (2025) 40:101–6. doi: 10.11847/zgggws1139802

[11] Xinxin Z Mengyu Z Yuying X Peng Z Anni C Xiuyu L . The level and factors associated with active health behavior capability among housebound disabled older adult. J Nurs Sci. (2025) 40:24–8. doi: 10.3870/j.issn.1001-4152.2025.16.101

[12] Yaacob LH Abdul Mokti S Muhammad J. Health seeking behaviour of menopausal women with urinary incontinence in north East Malaysia. J Women Aging. (2020) 32:537–45. doi: 10.1080/08952841.2019.1593799, 30967095

[13] Latifi M Alishan Karami N Miraj S Allahbakhshian L Rahimi F Karbasi A. Health information-seeking behavior of menopausal women: an online survey of Iranian menopausal women. Iran J Nurs Midwifery Res. (2025) 30:544–52. doi: 10.4103/ijnmr.ijnmr_27_24, 40832500 PMC12360755

[14] Gebretatyos H Ghirmai L Amanuel S Gebreyohannes G Tsighe Z Tesfamariam EH. Effect of health education on knowledge and attitude of menopause among middle-age teachers. BMC Womens Health. (2020) 20:232. doi: 10.1186/s12905-020-01095-2, 33046075 PMC7552533

[15] Xiao CQ Mou CW Zhou X. Effect of mindfulness meditation training on anxiety, depression and sleep quality in perimenopausal women. J South Med Univ. (2019) 39:998–1002. doi: 10.12122/j.issn.1673-4254.2019.08.19, 31511223 PMC6765589

[16] Zhao Y Niu H Liu S. Effects of aerobics training on anxiety, depression and sleep quality in perimenopausal women. Front Psych. (2022) 13:1025682. doi: 10.3389/fpsyt.2022.1025682, 36506429 PMC9730414

[17] Xu H Liu J Li P Liang Y. Effects of mind-body exercise on perimenopausal and postmenopausal women: a systematic review and meta-analysis. Menopause. (2024) 31:457–67. doi: 10.1097/GME.0000000000002336, 38669625 PMC11465887

[18] Zimet GD Dahlem NW Zimet SG Farley GK. The multidimensional scale of perceived social support. J Pers Assess. (1988) 52:30–41. doi: 10.1207/s15327752jpa5201_2, 2280326

[19] Yuan S Ren J. Social support and its influencing factors among perimenopausal women in Tianjin, China: a community-based study. Health Care. (2025) 13:1057. doi: 10.3390/healthcare13091057, 40361835 PMC12071810

[20] Acoba EF. Social support and mental health: the mediating role of perceived stress. Front Psychol. (2024) 15:1330720. doi: 10.3389/fpsyg.2024.1330720, 38449744 PMC10915202

[21] Zheng X Xue Y Dong F Shi L Xiao S Zhang J . The association between health-promoting-lifestyles, and socioeconomic, family relationships, social support, health-related quality of life among older adults in China: a cross sectional study. Health Qual Life Outcomes. (2022) 20:64. doi: 10.1186/s12955-022-01968-0, 35443689 PMC9022255

[22] Pearlin LI. The sociological study of stress. J Health Soc Behav. (1989) 30:241–56. doi: 10.2307/2136956 2674272

[23] Lai M-F Lin C-Y Griffiths MD Hou W-L. Emotional blackmail and depression among Taiwanese nurses: a cross-sectional study using the stress process model with coping strategies as mediator and social support as moderator. J Psychiatr Ment Health Nurs. (2026) 33:120–30. doi: 10.1111/jpm.70067, 41288233

[24] Schunk DH DiBenedetto MK. Motivation and social cognitive theory. Contemp Educ Psychol. (2020) 60:101832. doi: 10.1016/j.cedpsych.2019.101832

[25] Beh E Lin J Leong G Loh PY Poh EJ Chan D. Digital health interventions in children and adolescents with type 1 diabetes mellitus and their impact on clinical and behavioral outcomes: scoping review. JMIR Mhealth Uhealth. (2026) 14:e79338. doi: 10.2196/79338, 41773664 PMC12954698

[26] Harlow SD Gass M Hall JE Lobo R Maki P Rebar RW . Executive summary of the stages of reproductive aging workshop +10: addressing the unfinished agenda of staging reproductive aging. Climacteric. (2012) 15:105–14. doi: 10.3109/13697137.2011.650656, 22338612 PMC3580996

[27] Chow S-C Shao J Wang H Lokhnygina Y. Sample Size Calculations in Clinical Research. Boca Raton: Taylor & Francis (2017).

[28] Nylund-Gibson K Garber AC Carter DB Chan M Arch DAN Simon O . Ten frequently asked questions about latent transition analysis. Psychol Methods. (2023) 28:284–300. doi: 10.1037/met0000486, 35834194

[29] Heinemann LAJ Potthoff P Schneider HPG. International versions of the menopause rating scale (MRS). Health Qual Life Outcomes. (2003) 1:28. doi: 10.1186/1477-7525-1-28, 12914663 PMC183844

[30] Wang XD Wang XL Ma H. Handbook of Mental Health Rating Scales (Revised Edition) [M]. Beijing: Chinese Mental Health Journal (1999) 1999:131–133.

[31] Wang QQ Guo XQ Niu XL. Development of the active health management scale for perimeno-pause women based on the capability, opportunity, and motivation-behaviour (COM-B) model. J Nurs Sci. (2025) 40:35–9. doi: 10.3870/j.issn.1001-4152.2025.07.035

[32] Wang D Song P Zhang Q Zhuang B. Urban-rural differences in influencing factors of depressive symptoms among Chinese perimenopausal women. Sci Rep. (2025) 15:41029. doi: 10.1038/s41598-025-24883-y, 41266681 PMC12635278

[33] Sun D Shao H Li C Tao M. An analysis of the main reasons that perimenopausal and postmenopausal women in China have for seeking outpatient treatment and factors influencing their symptoms: a single-center survey. Clin Exp Obstet Gynecol. (2015) 42:146–51. doi: 10.12891/ceog1744.2015, 26054107

[34] Preacher KJ Hayes AF. SPSS and SAS procedures for estimating indirect effects in simple mediation models. Behav Res Methods Instrum Comput. (2004) 36:717–31. doi: 10.3758/BF03206553, 15641418

[35] Jiang M Chen L Zheng X Feng Y. Latent profile analysis of symptoms of depression and anxiety among perimenopausal women and their predictors. Front Psych. (2025) 16:1572570. doi: 10.3389/fpsyt.2025.1572570, 40534910 PMC12174408

[36] Xu H Zhang Z Ye Y Zhou J Cao X. Linking socioeconomic status to depressive symptoms in aging populations. Nat Ment Health. (2025) 3:1519–31. doi: 10.1038/s44220-025-00533-0

[37] Baquedano Mainar L Sanchez Mendez S Dieste Perez P Hernandez Aragon M Ladron de Guevara NM. Women’s knowledge about the genitourinary syndrome of menopause: adherence to its treatments in the COVID-19 era in a sample of them: COMEM-GSM study. Bmc Womens Health. (2021) 21. doi: 10.1186/s12905-021-01548-2 PMC8631561 34847892

[38] Tariq B Phillips S Biswakarma R Talaulikar V Harper JC. Women’s knowledge and attitudes to the menopause: a comparison of women over 40 who were in the perimenopause, post menopause and those not in the peri or post menopause. BMC Womens Health. (2023) 23:460. doi: 10.1186/s12905-023-02424-x, 37648988 PMC10469514

[39] Khademi K Kaveh MH Nazari M Asadollahi A. Effect of health model-based menopausal self-care training on quality of life and health: a randomised controlled trial. Health Expect. (2025) 28:e70511. doi: 10.1111/hex.70511, 41316699 PMC12662812

[40] Periyasamy U Ethiraj T Rajendran SS Tamilselvi P Elumalai G Subbaiya S . Impact of nurse-led digital education on perimenopausal women’s perception and practices for osteoporosis prevention. J Pharm Bioallied Sci. (2025) 17:S682–4. doi: 10.4103/jpbs.jpbs_1622_24, 40511143 PMC12156660

[41] Zhong X Gao K Zhang Y Hu Y Chen L Cai W . Women’s self-management of urinary incontinence: a qualitative study using the proactive health behavior framework. Int Urogynecol J. (2026) 37:2105–22. doi: 10.1007/s00192-025-06502-6, 41569412

[42] Leitao M Perez-Lopez FR Maroco J Pimenta F. Exploring weight management beliefs during the menopausal transition (ME-WEL project): a qualitative comparative study based on health belief model. Br J Health Psychol. (2025) 30:12779. doi: 10.1111/bjhp.12779, 39789891

[43] Cortés YI Harris LK Cazales A Marginean V Long C Woods-Giscombé CL . Feasibility and acceptability of menopausia, salud, corazón (menopause, health, heart): a pilot randomized trial to reduce cardiovascular disease risk in midlife Latinas. Menopause. (2026) 33:641–52. doi: 10.1097/GME.0000000000002718 41632258

[44] Elgzar WT Nahari MH Sayed SH Ibrahim HA. Determinant of osteoporosis preventive behaviors among perimenopausal women: a cross-sectional study to explore the role of knowledge and health beliefs. Nutrients. (2023) 15:3052. doi: 10.3390/nu15133052, 37447378 PMC10346160

[45] Khandehroo M Salary M Mahdizadeh M Peyman N. Educational intervention and menopausal adapting: a randomized controlled trial. Health Sci Rep. (2024) 7:e70298. doi: 10.1002/hsr2.70298, 39737393 PMC11682967

[46] Zhang Z YuanyuanWang Xie Z. Depressive symptoms trajectories among Chinese menopause transition women and their subsequent impact on life satisfaction: a longitudinal study based on the CHARLS database. Int J Gynaecol Obstet. (2026) 174:235–43. doi: 10.1002/ijgo.70826 41568418

[47] Jahnke HR Brinson A Fine SS Henrich N. Reduction in menopause symptoms after engagement in a digital health program. Digit Health. (2025) 11:20552076251395567. doi: 10.1177/20552076251395567, 41229935 PMC12603014

[48] Lu J Yuan Y Wei S Li K Tu S Xie L. The relationship between social support and health-promoting lifestyles among perimenopausal women: the mediating role of psychological resilience. BMC Womens Health. (2026) 26:104. doi: 10.1186/s12905-025-04258-1, 41566309 PMC12908352

[49] Ren J Zhu Y Lin J Fang J Huang R. Exploring the mechanism of self-management: the role of social support, self-efficacy, and health literacy based on meta-analytic structural equation modeling. BMC Public Health. (2025) 26:128. doi: 10.1186/s12889-025-24860-0, 41345933 PMC12797755

[50] Han J Tang R Zhang Z. Why do Chinese patients avoid health information? The role of caregiver burden perception and self-stigma. Patient Educ Couns. (2025) 140:109275. doi: 10.1016/j.pec.2025.109275, 40729980

[51] Magistro D Vagnetti R Ansdell P Piasecki J. Self-efficacy, quality of life, physical activity and educational interventions in menopausal women: a cross-sectional and pre-post study using bayesian structural equation modelling. Maturitas. (2025) 203:108774. doi: 10.1016/j.maturitas.2025.108774, 41252865

[52] de B Jaeger M Mina CS Alves S Schuh GJ Wender MC Manfro GG. Negative affect symptoms, anxiety sensitivity, and vasomotor symptoms during perimenopause. Braz J Psychiatry. (2021) 43:277–84. doi: 10.1590/1516-4446-2020-0871, 33053044 PMC8136388

[53] Abbas J Aqeel M Abbas J Shaher B Sundas J Zhang W. The moderating role of social support for marital adjustment, depression, anxiety, and stress: evidence from Pakistani working and nonworking women. J Affect Disord. (2019) 244:231–8. doi: 10.1016/j.jad.2018.07.071, 30173879

[54] Faleschini S Millar L Rifas-Shiman SL Skouteris H Hivert M-F Oken E. Women’s perceived social support: associations with postpartum weight retention, health behaviors and depressive symptoms. BMC Womens Health. (2019) 19:143. doi: 10.1186/s12905-019-0839-6, 31752823 PMC6873672

[55] Kamp KJ West P Holmstrom A Luo Z Wyatt G Given B. Systematic review of social support on psychological symptoms and self-management behaviors among adults with inflammatory bowel disease. J Nurs Scholarsh. (2019) 51:380–9. doi: 10.1111/jnu.12487, 31119856

[56] Racodon M Duvilliers V. The impact of health education on the quality of life of menopausal and perimenopausal women: a systematic literature review. Gynecol Obstet Fertil Senol. (2026) 54:32–40. doi: 10.1016/j.gofs.2025.10.011, 41077403

[57] Zhang Z Xu H Zhou J Cao X. China’s healthy city pilot policy improves physical and mental health outcomes for middle-aged and older adults: a quasi-natural experiment based on CHARLS. Int J Geriatr Psychiatry. (2025) 40:e70082. doi: 10.1002/gps.70082, 40280890

