# Supplementary material for: The relationship between proactive health management, perceived social support, and symptoms in perimenopausal women: a latent profile and mediation analysis
Source: Front Public Health. 2026 Sep 29;14:1889102. doi: 10.3389/fpubh.2026.1889102 (PMC13658004; doi:10.3389/fpubh.2026.1889102)
Supplement: Supplementary file 1 [file Table_1.docx]

**Supplementary Material**

**Table S1** The multifactor analysis of proactive health management by logistic regression (N = 491)

|  | C1 VS C3 | |  | C2 VS C3 | |  | C2 VS C1 | |
| --- | --- | --- | --- | --- | --- | --- | --- | --- |
| Variable | P | OR（95%CI） |  | P | OR（95%CI） |  | P | OR（95%CI） |
| Age(ref=56-60 years) |  |  |  |  |  |  |  |  |
| 40 years and above | 0.824 | 0.881(0.289-2.686） |  | 0.748 | 1.218(0.367-4.038) |  | 0.572 | 1.382(0.450-4.246) |
| 46 years and above | 0.720 | 1.202(0.439-3.292) |  | 0.342 | 1.72(0.562-5.266) |  | 0.483 | 1.430(0.526-3.887) |
| 51 years and above | **0.012** | 0.340(0.146-0.791) |  | 0.267 | 1.699(0.666-4.333) |  | **＜0.001** | 4.994(2.175-11.464) |
| Education Level(ref= Bachelor's degree or above) | | | | | | | | |
| High school or below | **0.018** | 3.272(1.222-8.762) |  | 0.216 | 1.88(0.692-5.110) |  | 0.362 | 0.575(0.175-1.890) |
| College | 0.163 | 2.369(0.705-7.956) |  | 0.303 | 1.891(0.563-6.358) |  | 0.760 | 0.798(0.189-3.376) |
| Residence(ref= Urban) |  |  |  |  |  |  |  |  |
| Rural | **0.038** | 1.771(1.031-3.042) |  | 0.591 | 1.172(0.657-2.093) |  | 0.137 | 0.662(0.384-1.140) |
| Chronic Diseases(ref=No) |  |  |  |  |  |  |  |  |
| Yes | **0.024** | 2.156(1.105-4.207) |  | 0.159 | 1.667(0.818-3.397) |  | 0.414 | 0.773(0.417-1.434) |
| Menstrual Status(ref= Early postmenopause) |  |  |  |  |  |  |  |  |
| Early perimenopause | **0.018** | 0.350(0.147-0.834) |  | 0.372 | 0.686(0.300-1.568) |  | 0.104 | 1.960(0.870-4.413) |
| Late perimenopause | 0.224 | 0.589(0.251-1.383) |  | **0.002** | 0.209(0.077-0.569) |  | **0.036** | 0.355(0.135-0.934) |
| Occupation(ref= Retired) |  |  |  |  |  |  |  |  |
| Manual laborer | **0.004** | 4.195(1.566-11.237) |  | **0.001** | 8.629(2.554-29.154) |  | 0.279 | 2.057(0.558-7.583) |
| On-duty staff | **＜0.001** | 7.670(2.485-23.679) |  | **0.005** | 6.852(1.772-26.496) |  | 0.878 | 0.893(0.210-3.793) |
| Self-employed | **＜0.001** | 8.221(2.673-25.284) |  | **0.001** | 10.324(2.693-39.583) |  | 0.749 | 1.256(0.311-5.077) |
| Professional/technical staff | 0.189 | 2.818(0.600-13.228） |  | 0.796 | 0.729(0.066-7.997) |  | 0.310 | 0.259(0.019-3.528) |
| Unemployed/looking for work | 0.828 | 0.868(0.243-3.099） |  | **0.006** | 6.701(1.737-25.848) |  | **0.009** | 7.717(1.662-35.831) |
| Full-time homemaker | **0.016** | 3.867(1.283-11.653) |  | **0.008** | 6.395(1.633-25.049) |  | 0.488 | 1.654(0.399-6.856) |
| Monthly Household Per Capita Income（ref=＞6000 RMB） | | | | | | | | |
| ＜4000 RMB | **＜0.001** | 6.328(3.170-12.634) |  | **0.004** | 2.963(1.412-6.216) |  | 0.051 | 0.468(0.219-1.002) |
| 4000-6000 RMB | **0.029** | 2.104(1.080-4.102） |  | **0.008** | 2.521(1.28-4.968) |  | 0.639 | 1.198((0.564-2.546) |

**Note:** Bold figures highlight statistically significant in the multifactor analysis. C1= Low-level passive group;C2=Low-ability high driven group;C3=High-level active health group.
